# Image Shadow Detection and Removal Based on Region Matching of Intelligent Computing

**DOI:** 10.1155/2022/7261551

**Published:** 2022-04-20

**Authors:** Junying Feng, Yong Kwan Kim, Peng Liu

**Affiliations:** ^1^School of Intelligent Manufacturing, Weifang University of Science and Technology, Shandong, Weifang 261000, China; ^2^Department of Information and Communication Engineering, Hoseo University, Chungcheongnam-do, Asan, 31499, Republic of Korea

## Abstract

Shadow detection and removal play an important role in the field of computer vision and pattern recognition. Shadow will cause some loss and interference to the information of moving objects, resulting in the performance degradation of subsequent computer vision tasks such as moving object detection or image segmentation. In this paper, each image is regarded as a small sample, and then a method based on material matching of intelligent computing between image regions is proposed to detect and remove image shadows. In shadow detection, the proposed method can be directly used for detection without training and ensures the consistency of similar regions to a certain extent. In shadow removal, the proposed method can minimize the influence of shadow removal operation on other features in the shadow region. The experiments on the benchmark dataset demonstrate that the proposed approach achieves a promising performance, and its improvement is more than 6% in comparison with several advanced shadow detection methods.

## 1. Introduction

Although the shadow in the image is a kind of image information, it often interferes with object recognition, image segmentation, and other image processing work. Therefore, the detection and removal of shadow have been widely concerned. The difficulty of shadow detection lies in the complexity of background and light in the image, as well as the interference of dark objects in the image. The difficulty of shadow removal is to ensure that only the shadow is removed without changing other image features in the shadow region.

Inspired by the relevant articles on shadow detection and removal through region matching and other methods [1, 2], this paper proposes a shadow detection and removal method based on interarea material matching without training. This method can accurately detect the shadow region and minimize the impact of shadow removal on other features in the shadow region.

The contributions of this paper are as follows. (1) In shadow detection, based on the mutual restriction between image region features and matching region, this method can detect directly without training. (2) In shadow removal, the shadow region is restored according to the region matching. The calculation time is very short. Other features in the shadow region can be kept unchanged to a great extent, and only the shadow is restored.

The remainder of this paper is organized as follows.  Section 2 introduces the related works of our proposed approach. In Section 3, we present the processes of shadow detection and removal. The experiments and experimental results' analysis are described in Section 4, and finally, this paper is briefly summarized in Section 5.

## 2. Related Works

At present, the commonly used shadow detection and removal methods are divided into three directions: the method based on a physical model, the method based on basic image features, and the method based on machine learning. At the same time, there are many ways to combine these directions.

### 2.1. Shadow Detection

#### 2.1.1. Shadow Feature

At present, shadow detection based on image features is widely studied, mainly to extract some changing and invariant features in shadow. Zhu et al. [3] proposed a variety of features that change and remain unchanged under the shadow in the gray image, including the features of distinguishing dark objects from shadow regions. Lalonde et al. [4] emphasized the local features of the image and determined the shadow boundary quickly by comparing the features of adjacent regions. Chung et al. [5] proposed a single feature threshold method for directly distinguishing shadow regions. Dong et al. [6] proposed the characteristics of brightness and change rate. Some color-related features were mentioned in [1, 7] to distinguish shadow regions.

#### 2.1.2. Machine Learning Method

Currently, a lot of works combined the direct distinction of original features with machine learning algorithms to train classifiers to distinguish shadow regions under the premise of data-driven [1, 3, 4, 8]. At the same time, for the models that distinguish each pixel separately, most of the works used CRF, MRF, and other similar models to maintain regional consistency [3, 9–11]. With the development of deep learning algorithms in recent years, deep learning models have also been applied to shadow detection [9, 10, 12]. Deep learning models make the original feature engineering no longer necessary, but these algorithms are often based on a large amount of data and need a long time for model training.

### 2.2. Shadow Removal

User assistance shadow removal can be completed under two conditions: automatic image shadow detection and input of some image information with user assistance. Different from the earlier user assistance that requires users to clearly input various complex information such as shadow contour [13–15], at present, some user assistance methods only need to input a small amount of information to obtain good results [16–18].

#### 2.2.1. Shadow Removal Algorithm

At present, many shadow removal algorithms are based on shadow matting [1, 9], that is, to decompose the image into the form of some parameters acting on the original unshaded image, and then remove the shadow by calculating these parameters. On the basis of shadow detection, shadow boundary and image reorganization with shadow can be used to remove shadow [19–21]. Vicente and Samaras [2] proposed a method to restore the shadow by using the adjacent region of the shadow region, but this method required the shadow region to have the same material as its adjacent region. At the same time, some recent machine learning algorithms and deep learning methods also have good performance in shadow removal [9, 12, 22].

## 3. The Proposed Method

**Figure 1:**
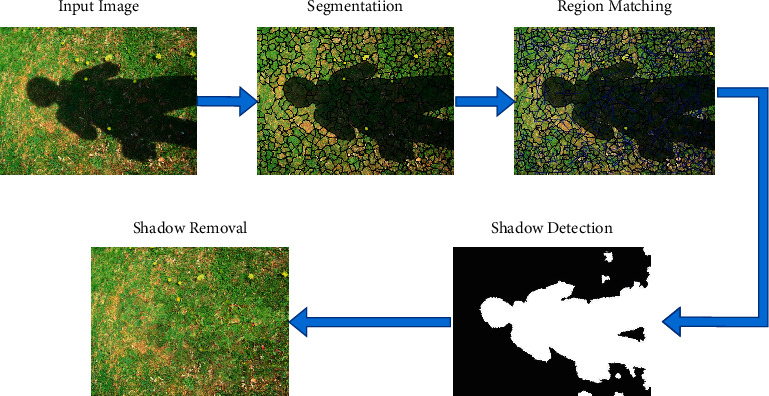
Pipeline of the proposed method.

The proposed method in this paper is based on image region matching and image optical features. Firstly, the Meanshift [23] algorithm is used to segment the image into a total of *n* regions. Each region is recorded as *S*_*i*_, and the center is *C*_*i*_. Then, match the region with the closest material for each region (see Section 3.1). Shadow detection (see Section 3.2) and shadow removal (see Section 3.3) are realized according to region matching. The overall pipeline is shown in Figure 1.

### 3.1. Region Matching Calculation

Since the color, saturation, and other features will change in the shadow region and the target is looking for the region with the most similar materials, the features used in region matching are the two shadow-invariant features mentioned in reference [3]: gradient feature and texture feature.

#### 3.1.1. Gradient Feature

The gradient change of the image region is hardly affected by shadow. The gradient similarity between regions with similar materials is stronger. In order to extract this feature, a histogram is calculated using the gradient value of each region in the image, and the similarity between regions is measured through the Euclidean distance of the histograms between two regions.

#### 3.1.2. Texture Feature

Image surface texture features are almost independent of shadows. Specifically, the method in reference [24] is used to extract texture features. Similarly, the similarity between regions is measured by calculating the Euclidean distance of the histograms of two regions.

#### 3.1.3. Distance between Regions

In order to ensure the local consistency, the distance between the central points of the two regions is added as the factor to judge the regional similarity. The calculation of similarity between regions is shown in the following equation:

(1) $$\begin{array}{c} D_{i,j}=D_{{\text{gradient}}_{i,j}}+D_{{\text{texture}}_{i,j}}+D_{{\text{distance}}_{i,j}}, \end{array}$$

where *D*_gradient_*i*,*j*__, *D*_texture_*i*,*j*__, and *D*_distance_*i*,*j*__, respectively, represent gradient feature similarity, texture feature similarity between regions *i*, *j*, and distance between center points of regions *i*, *j*.

### 3.2. Shadow Detection

In shadow detection, on the one hand, the shadow is judged by the shadow features; on the other hand, the shadow is judged by the mutual restriction between regions.

#### 3.2.1. Feature Selection

In color images, color tone is a powerful descriptor that simplifies and dominates feature identification and extraction in visual pattern recognition applications. Shadow detection and removal require separate chrominance and luminance information. Since the image represented by the RGB color space is a mixture of chrominance and luminance information into the three components of R, G, and B, we need some other color models to handle shadow. There are five invariant color models, HSI model, HSV model, HCV model, YIQ model, and YCbCr model. All of the models consist of independent chromaticity and luminance. We select three common color space models to detect and remove shadows.

(1) Y-channel information in YCbCr color space: convert the original image from RGB color space to YCbCr color space. According to the method mentioned in reference [25], when the value of a pixel on the Y-channel in YCbCr space is less than 60% of the average value of the Y-channel of the whole image, it can be directly considered that the pixel is in shadow. In the algorithm introduced in Section 3.2.2, when the average value of the Y-channel of a region is less than 60% of the average value of the Y-channel of the whole image, this region is considered in the shadow. Take the average value from the region *S*_*i*_ and record the feature as *Y*_*i*_.
(2) HSI color space information: convert the original image from RGB space to HSI space and then normalize the values on H and I channels to the [0, 1] interval to obtain *H*_*e*_ and *I*_*e*_. According to the method in reference [5], the value in equation (2) is extracted for each pixel as another feature of the image. Take the average value from the region *S*_*i*_ and record the feature as *R*_*i*_.
  (2) $$\begin{array}{c} R\left(x,y\right)=\frac{H_{e}\left(x,y\right)+1}{I_{e}\left(x,y\right)+1}. \end{array}$$
*R*(*x*, *y*), *H*_*e*_(*x*, *y*), and *I*_*e*_(*x*, *y*) represent pixel at position (x, y) in *R*, image *H*_*e*_, and image *I*_*e*_, respectively.
(3) HSV color space information: convert the original image from RGB space to HSV color space. We utilize the chrominance channel H as the other feature. Take the average value from the region *S*_*i*_ and record the feature as *H*_*i*_.

#### 3.2.2. The Proposed Algorithm

**Figure 2:**
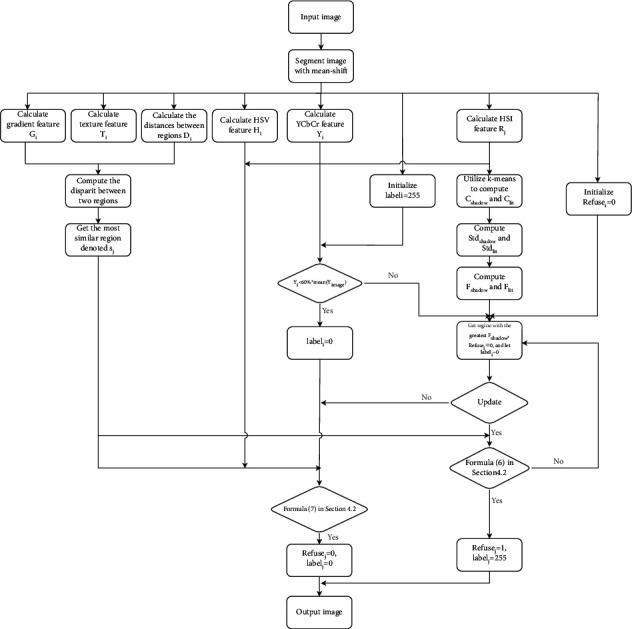
The flowchart of the shadow detection.

Figure 2 shows the flowchart of the shadow detection approach.

The specific implementation steps of the proposed shadow detection method are as follows.

 
*Variable Preparation.*
(1) Segment image with Meanshift algorithm and then obtain *n* regions; each region is remarked as *S*_*i*_. The center of each region is *C*_*i*_.
(2) Compute the disparity between *S*_*i*_ and *S*_*j*_ and get the corresponding region with the highest similarity for a given region which is denoted as Near_*i*_. At the same time, record the information label_*i*_ of whether the region *i* is in shadow and initialize label_*i*_ to 255. The similarity of regions *i* and *j* is computed as equation (1).
(3) For all *R*_*i*_, 1 < *i* < *n*, use K-means algorithm to calculate two centers *C*_shadow_ and *C*_*lit*_ which, respectively, represent whether it is the feature center of the shadow region. Suppose *R* follows a normal distribution; the standard deviations Std_shadow_ and Std_*lit*_ are calculated, which correspond to *C*_shadow_ and *C*_*lit*_, respectively. Then, for every *R*_*i*_, *F*_shadow_ and *F*_*lit*_ corresponding to *C*_shadow_ and *C*_*lit*_ are computed.
(4) For each region *S*_*i*_, Refuse_*i*_ represents whether *S*_*i*_ is forbidden to be a shadow region because of other regions, and Refuse_i_ is initialized to 0.
 
*Steps*.
(1) Extract features *Y*_*i*_ and *R*_*i*_ and prepare relevant variables.
(2) If *Y*_*i*_ < 60%*∗*mean(*Y*_image_), label_*i*_=shadow.
(3) Select the region *S*_*i*_ with the greatest *F*_shadow_ and Refuse_*i*_=0, and set label_*i*_=shadow.
(4) Denote the nearest region Near_*i*_ of *S*_*i*_ as *S*_*j*_. Check whether *S*_*i*_ and *S*_*j*_ are brightness opposite regions by comparing *R*_*i*_ and *R*_*j*_, and if so, then judge Refuse_*j*_=1.
(5) Iteratively execute steps (3) and (4) until no update.
(6) For the region *S*_*i*_ of label_*i*_=shadow, if the brightness of *S*_*i*_ is similar to *S*_*j*_ and Refuse_*j*_=0 by comparing *Y*_*i*_, *Y*_*j*_, *R*_*i*_, *R*_*j*_, set label_*j*_=0.

### 3.3. Shadow Removal

**Figure 3:**
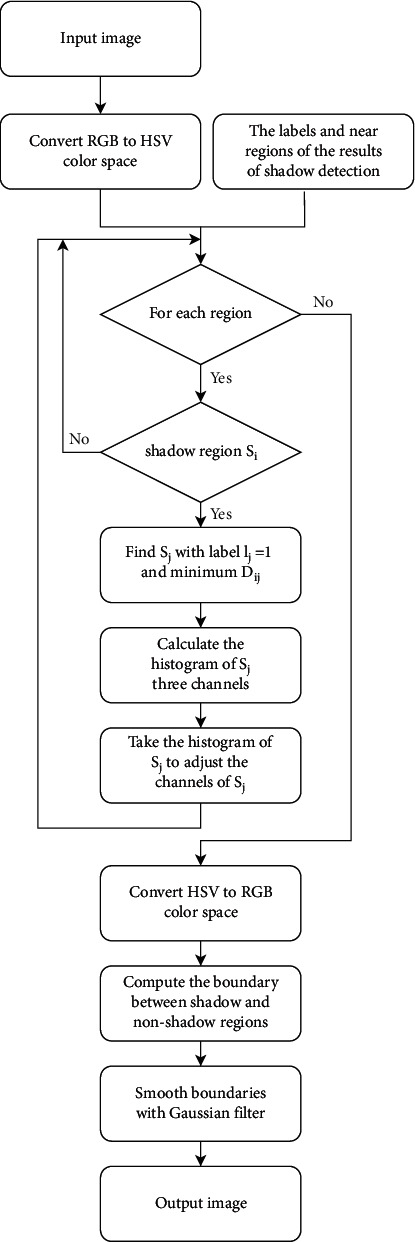
The flowchart of the shadow removal.

Shadow removal is mainly carried out in HSV color space. The overall idea is to find the corresponding region *S*_*j*_ for the shadow region *S*_*i*_ to meet label_*j*_=1 and minimize *D*_*i*,*j*_. Therefore, *S*_*j*_ is the most similar non-shaded region of *S*_*i*_. Consider using *S*_*j*_ to adjust the brightness of *S*_*i*_, remove the shadow on the *S*_*i*_ through the histogram matching algorithm, and minimize the impact of the operation on other features. The flowchart of shadow removal is shown in Figure 3.

#### 3.3.1. Histogram Matching

Supposing the feature of region *S*_*i*_ as Feature_*i*_, given template histogram Hist_*T*_, the purpose of histogram matching is to ensure that the overall offset conforms to the distribution of the template *T* under the condition of ensuring the minimum change of mutual distributions between Feature_*i*_. The specific methods are as follows.

1. Calculate the histogram Hist_*i*_ of Feature_*i*_ with the number of bins equal to *T*.
2. The cumulative histograms *Acc*_*i*_ and *Acc*_*T*_ are calculated for Hist_*i*_ and Hist_*T*_, respectively.
3. For each stripe *p* in *Acc*_*i*_, calculate the bin number *q* with the smallest interpolation among *Acc*_*i*_.
4. Move each bin *p* to the position *q*.

#### 3.3.2. The Proposed Algorithm

The specific implementation steps of the proposed shadow removal method are as follows.

1. Calculate the shadow detection result label and convert the image to HSV space at the same time.
2. Repeat steps (3)–(5) for each shadow region *S*_*i*_.
3. For region *S*_*i*_, find *S*_*j*_ with label_*j*_=1 and minimum *D*_*i*,*j*_, and use *S*_*j*_ to relight *S*_*i*_.
4. In the HSV color space, compute the histograms Hist_*H*,*j*_, Hist_*S*,*j*_ and Hist_*V*,*j*_ of S_j_ in the three channels H, S, and V, respectively..
5. Take Hist_*H*,*j*_, Hist_*S*,*j*_, Hist_*V*,*j*_ as the template of histogram matching and adjust the three features of *S*_*i*_, so that the feature distribution is close to the template.
6. Convert the image to RGB space.
7. Calculate the intersection boundary of all shadow regions and non-shaded regions in the image and then smooth all boundaries using a Gaussian filter.

## 4. Experiments

Experiments are conducted on the shadow dataset SBU [26]. SBU dataset is a large shadow detection dataset, which contains 4727 images, and each image has a ground truth based on pixel markers. In order to quantitatively evaluate the effectiveness of the proposed method and comparative methods, the commonly used indexes such as recall, specificity, and balanced error rate (BER) are used as the evaluation indexes, which are defined as follows:

(3) $$\begin{array}{c} \text{recall}=\frac{\text{TP}}{\text{TP}+\text{FN}},\\ \text{specificity}=\frac{\text{TN}}{\text{TN}+\text{FP}},\\ \text{BER}=1-0.5*\left(\text{recall}+\text{specificity}\right), \end{array}$$

where *TP* indicates that the real shadow is correctly classified, *FN* indicates that the real shadow is incorrectly classified as non-shaded, *TN* indicates that the non-shaded pixels are correctly classified, and *FP* indicates that the non-shaded pixels are incorrectly classified as shadow pixels. The higher the recall is, the more the shadow pixels are found. The higher the specificity is, the more the non-shaded pixels are recalled. Recall and specificity are considered a pair of contradictions. The comprehensive performance of the algorithm is tested by BER. The smaller the BER, the better the shadow detection performance.

### 4.1. Region Matching

**Figure 4:**
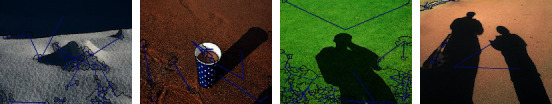
Most similar region matching.

Region matching is shown in Figure 4. The blue line in the figure connects the center of each pair of matching regions. It can be seen that when the image segmentation is more accurate, the nearest region matching is also more accurate.

### 4.2. Shadow Detection

The parameter setting of shadow detection is mainly in steps (4) and (6) of Section 3.2.2.

In step (4), when equation (4) is satisfied between *S*_*i*_ and *S*_*j*_, it is considered that the label properties of *R*_*i*_ and *R*_*j*_ are opposite, and set Refuse_*j*_=1.

(4) $$\begin{array}{c} \frac{R_{i}-C_{\text{shadow}}}{{\text{Std}}_{\text{shadow}}}-\frac{R_{j}-C_{\text{shadow}}}{{\text{Std}}_{\text{shadow}}}>3, \end{array}$$

where *R*_*i*_ and *R*_*j*_ are the features of shadow detection in the region *S*_*i*_ and *S*_*j*_. The value of *R*_*i*_ ado *R*_*j*_ can be computed by equation (2). *C*_shadow_ is the feature center of the shadow areas, and Std_shadow_ is the standard deviation corresponding to the *C*_shadow_. Step (3) of Section 3.2.2 describes the details.

In step (6), when equation (5) is satisfied between *S*_*i*_ and *S*_*j*_, it is considered that the label properties of *S*_*i*_ and *S*_*j*_ are the same, and set label_*j*_=0.

(5) $$\begin{array}{c} \frac{\text{min }\left(H_{i},H_{j}\right)}{\text{max }\left(H_{i},H_{j}\right)}+\frac{\text{min }\left(Y_{i},Y_{j}\right)}{\text{max }\left(\left(Y_{i},Y_{j}\right)\right.}+\frac{\text{min }\left(R_{i},R_{j}\right)}{\text{max }\left(R_{i},R_{j}\right)}>2.5, \end{array}$$

where *H*_*i*_, *Y*_*i*_, *R*_*i*_, *H*_*j*_, *Y*_*j*_, and *R*_*j*_ are the shadow detection features of regions *S*_*i*_ and *S*_*j*_, respectively.

3 and 2.5 are empirical constants. Tuning of the parameters is outside the scope of the current work.

The performance of the proposed shadow detection method is evaluated by comparing it with two commonly used methods: Unary-Pairwise [27] and Stacked-CNN [26]. The Unary-Pairwise method is one of the best statistical methods to detect shadows from a single image. The Stacked-CNN method is a shadow detection method based on the deep learning framework using shadow prior map.

#### 4.2.1. Qualitative Analysis

**Figure 5:**
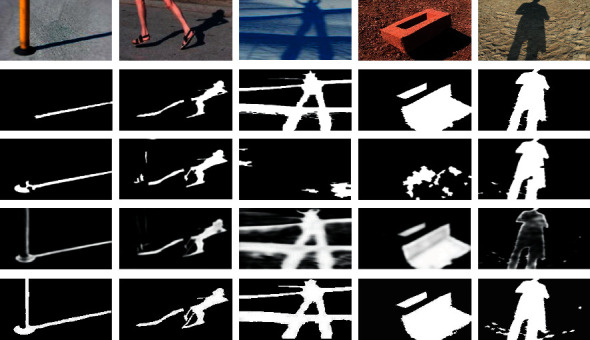
Qualitative comparison of the proposed method with other methods. Rows from top to bottom: input images, ground truths, results of the Unary-Pairwise method, results of the Stacked-CNN method, and results of the proposed method.

Figure 5 shows the shadow detection results of some images in the SBU dataset.

It can be seen from the third line of Figure 5 that the Unary-Pairwise method has good shadow detection results in some images, but almost no shadow is detected in columns 3 and 4. The fourth line of Figure 5 shows the shadow detection results of the Stacked-CNN method, which can detect all correct shadows, but the visual effects of some results are blurred.

As can be seen from the last line of Figure 5, the proposed method has relatively small error in image shadow detection, but the detection is basically correct. The consistency of shadow regions is ensured by region matching. It should be noted that in the images in column 1, the black base of the pole is detected as a shadow, which is caused by the similar color features between the black base and the shadow. Objects with color features similar to shadow will reduce the precision of shadow detection.

#### 4.2.2. Quantitative Analysis

Table 1 shows the results of quantitative analysis on the SBU dataset. The proposed method can improve the detection rate of shadow and non-shaded regions. Obviously, recall and specificity are considered a pair of contradictions. Considering the performance of shadow detection, the BER metric is used, and the proposed method effectively reduces the BER.

The Stacked-CNN method can detect most shadow pixels in the dataset, but it also causes pixels in non-shaded areas to be insufficiently recalled. Some non-shaded pixels are wrongly classified as shadow pixels, resulting in blurred shadow detection images, which is consistent with the results of qualitative analysis. The Unary-Pairwise method has the lowest recall of shadow pixels on a large-scale shadow detection dataset. This is because of the false pairing between regions due to the loose constraints. Although the recall of the proposed method is lower than that of the Stacked-CNN method, the precision is higher. On average with BER, the improvement made by the proposed method is more than 6% which is impressive.

In Table 2, we show the execution time of testing phases of the proposed approach. Our method does not need training, and it works faster than the statistical method and one order of magnitude faster than the deep learning method.

### 4.3. Shadow Removal

The parameters of shadow removal are set in step (7) of Section 3.3.2, in which Gaussian blur is applied to the points whose distance from the edge pixel is less than or equal to 2, and the parameters of Gaussian blur are set to *h*size=15, *σ*=15.

**Figure 6:**
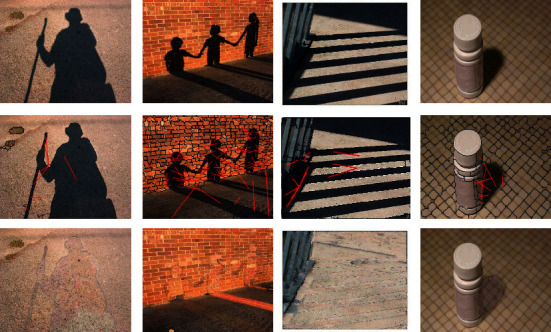
Qualitative analysis results of the proposed shadow removal method on the SBU dataset. Rows from top to bottom: input images, shadow removal matching regions, and shadow removal images.

The second line of Figure 6 shows the shadow removal process of some images in SBU dataset, in which the red lines connect and match the corresponding shadow region *S*_*i*_ and non-shaded region *S*_*j*_. Use *S*_*j*_ to relight *S*_*i*_ through the algorithm in Section 3.3.2.

The third line of Figure 6 shows the shadow removal results of the images. It can be seen that the regions after removing shadow through region matching maintain the original texture features, but the brightness changes. The image after removing shadow has an obvious boundary around the original shadow region, but the edge effect is significantly weakened after Gaussian smoothing.

**Figure 7:**
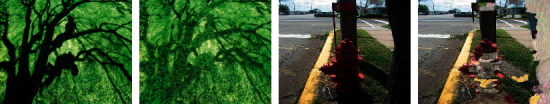
Failed images for shadow removal on the dataset.

Through experiments, it is found that not all the shadows of the image can be removed correctly. Two failed shadow removal scenarios are shown in Figure 7. In the first image, the shadow occupies most of the image, and the shadow is evenly distributed in the image, which will lead to the uniform distribution of image feature histogram, and the non-shaded region is not enough to remove the shadow region. In the second image, the shadows of multiple objects are complex, and the fire hydrant is almost completely in shadow, resulting in the wrong shadow removal matching, so the shadows cannot be removed correctly.

In this work, the experiments are run on a PC with a 3.6 GHz CPU and 8G RAM in the Matlab 2018b environment under Windows 10.

## 5. Conclusion

Experiments show that the shadow detection and removal algorithm based on region matching is effective. In the aspect of shadow detection, the consistency of images can be guaranteed to a certain extent through the mutual restriction of regions with the same material; in the aspect of shadow removal, the method of brightening the shadow by the region with similar material can minimize the impact of shadow removal on the non-shaded features in the shadow region.

The limitation of the method proposed in this paper is that it is difficult to ensure the accuracy of region matching for complex images. Therefore, in shadow detection and shadow removal, it will bring wrong constraints due to wrong matching or restore the region to the wrong material. At the same time, this algorithm is based on Meanshift image segmentation technology, which is difficult to segment accurately in complex images.

The further improvement mainly lies in the calculation of the material matching region. First, using more and more datasets, the matching region is given by training classifiers and other methods. Second, adding more shadow-invariant features to improve the accuracy of region matching in complex images will be taken into account.

## Figures and Tables

**Table 1:** Evaluation of shadow detection methods on SBU dataset.

Method	Recall	Specificity	BER
Unary-Pairwise	0.5636	0.9357	0.2504
Stacked-CNN	**0.8609**	0.9059	0.1166
Our method	0.8460	**0.9361**	**0.1090**

The bold entries indicate the best result in a given column.

**Table 2:** Time complexity of shadow detection methods on the SBU dataset.

Method	Testing (hours)	Testing (sec/image)	Training (hours)
Unary-Pairwise	9.13	51.56	∼
Stacked-CNN	25.08	141.56	9.4
Our method	**3.08**	**17.40**	∼

The bold entries indicate the best result in a given column.

## Data Availability

The data used to support the findings of this study are included within the article.
